# MZB1 enables efficient interferon α secretion in stimulated plasmacytoid dendritic cells

**DOI:** 10.1038/s41598-020-78293-3

**Published:** 2020-12-14

**Authors:** Tanya Kapoor, Mauro Corrado, Erika L. Pearce, Edward J. Pearce, Rudolf Grosschedl

**Affiliations:** 1 Department of Cellular and Molecular Immunology, Max Planck Institute of Immunobiology and Epigenetics, 79108 Freiburg, Germany; 2 Department of Immunometabolism, Max Planck Institute of Immunobiology and Epigenetics, 79108 Freiburg, Germany

**Keywords:** Cell biology, Immunology

## Abstract

MZB1 is an endoplasmic reticulum (ER)-resident protein that plays an important role in the humoral immune response by enhancing the interaction of the μ immunoglobulin (Ig) heavy chain with the chaperone GRP94 and by augmenting the secretion of IgM. Here, we show that MZB1 is also expressed in plasmacytoid dendritic cells (pDCs). *Mzb1*^*−/−*^ pDCs have a defect in the secretion of interferon (IFN) α upon Toll-like receptor (TLR) 9 stimulation and a reduced ability to enhance B cell differentiation towards plasma cells. *Mzb1*^*−/−*^ pDCs do not properly expand the ER upon TLR9 stimulation, which may be accounted for by an impaired activation of ATF6, a regulator of the unfolded protein response (UPR). Pharmacological inhibition of ATF6 cleavage in stimulated wild type pDCs mimics the diminished IFNα secretion by *Mzb1*^*−/−*^ pDCs. Thus, MZB1 enables pDCs to secrete high amounts of IFNα by mitigating ER stress via the ATF6-mediated UPR.

## Introduction

Plasmacytoid dendritic cells (pDCs) were initially discovered in human lymph nodes in the 1950′s^1^. Their morphology proved to be controversial, with a lymphoid appearance that changes to a dendritic cell-like upon activation. The plasma cell-like appearance led to their nomenclature as “Plasmacytoid dendritic cells”^2^. The defining feature of pDCs is their ability to produce high levels of Type I Interferon (IFN) in response to viruses and viral components that are recognized by endosomal innate immune receptors such as the Toll-like receptors (TLR) 7 and TLR9^3,4^. Upon stimulation of the TLRs with an agonist, 60% of the pDCs transcriptome is dedicated to the expression and secretion of IFNα^5^, whereby each cell is capable of producing a staggering 1–3 picograms within 24 h^5,6^. Due to their high IFN production, pDCs play an important role in controlling the initial stage of viral infections. Secreted IFNα enhances the activation of NK cells, B and T cells^3^. Moreover, pDCs have also been found to be a major source of IFNα in inflammatory autoimmune diseases such as Systemic Lupus Erythematosus (SLE), psoriasis and Scleroderma^7–9^. As highly secretory cells, pDCs and plasma cells are the only hematopoietic cell type that depend on an augmented protein-folding ability of the endoplasmic reticulum (ER) for their survival^10,11^.

Accumulation of unfolded proteins in the ER results in an ER stress that can be alleviated by the activation of the unfolded protein response (UPR), which restores cellular proteostasis^12^. In particular, secretory cells require an enhanced protein-folding capacity of the ER which involves the sensing of ER stress, an increased expression of protein folding chaperones and the expansion of the ER. The UPR consists of three pathways, the inositol-requiring transmembrane kinase/endonuclease (IRE1), the PKR-like ER protein kinase (PERK) and the activating transcription factor 6 (ATF6) pathways^12–14^. Upon accumulation of misfolded proteins, BiP dissociation leads to the activation of these three transmembrane proteins^15^. Activation of IRE1 generates an active isoform of the transcription factor XBP1 that can dimerize with the activated isoform of ATF6 which is generated by its translocation to the Golgi and proteolytic cleavage^15–19^. ATF6 plays a crucial role in increasing levels of the ER chaperones BiP and GRP94 to help cope with the increased folding demands. Moreover, ATF6 and XBP1 regulate ER expansion by increased lipid biosynthesis, whereby ER membrane expansion can alleviate ER stress independent of an accompanying increase in ER chaperone levels^20,21^. A fine balance is maintained between the different pathways, whereby IRE1 and ATF6 are the protective ones that are activated early upon ER stress and mediate protein folding^22^. Upon continued unmitigated stress and failure to restore protein homeostasis the PERK pathway plays a more prominent role, leads to the expression of CHOP, which is involved in ER associated protein degradation (ERAD)^23,24^.

The ER resident protein MZB1 (pERP1) is abundantly expressed in innate-like B cells, including Marginal zone (Mz) B cells of the spleen and peritoneal B1 B cells, as well as in antibody-secreting cells^25–27^. Deletion of *Mzb1* resulted in a reduced humoral response with plasma cells secreting lower levels of IgM following in vivo stimulation of B cells^28,29^. Proteomic analysis of MZB1 interaction partners identified many proteins involved in the UPR, including the chaperones BiP, GRP94 (Hsp90B1), the calcium-binding proteins calnexin and calreticulin and the protein disulphide isomerase ERp57 and PDIA6^25^. MZB1 acts as a co-chaperone of GRP94 that is needed for its interaction with various client proteins, including immunoglobulins and integrins^28–30^. Recent transcriptome analysis of all hematopoietic cell types showed that *Mzb1* mRNA is also expressed in the non-B cell lineage cell the pDCs^31^. Here, we examine the function of MZB1 in pDCs and find that the deletion of *Mzb1* results in reduced IFNα upon TLR9-mediated stimulation. We show that this defect involves an impaired activation of the ER stress sensor ATF6 and a diminished expansion of the ER upon cell stimulation by TLR9.

## Results

### MZB1 is expressed in plasmacytoid dendritic cells and regulates IFNα secretion

**Figure 1:**
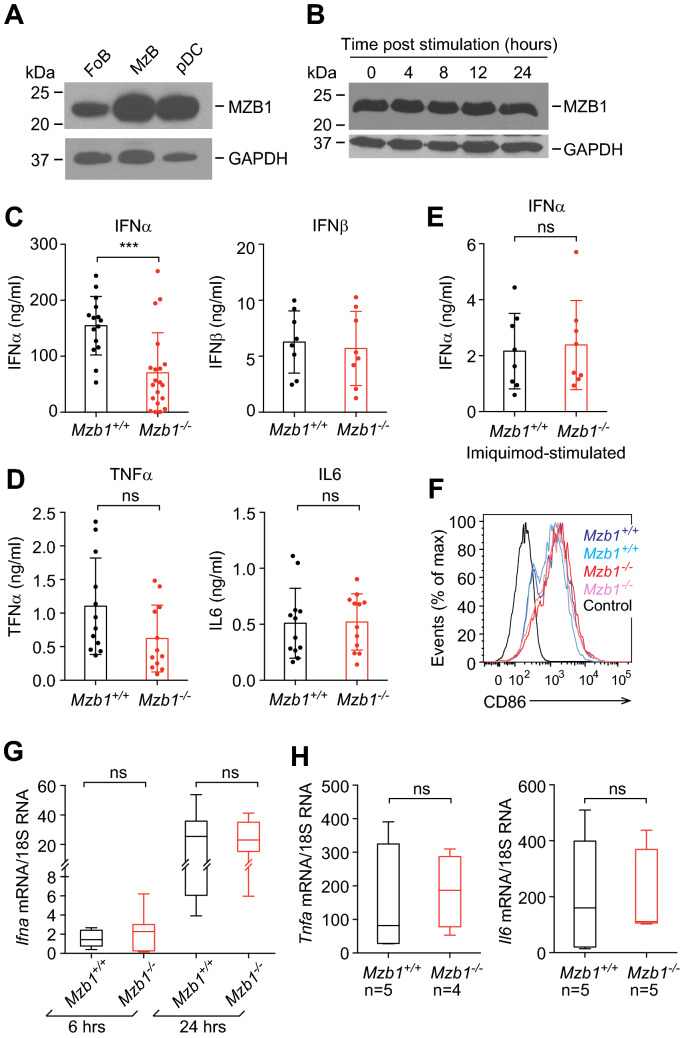
MZB1 is expressed in pDCs and inactivation of *Mzb1* affects IFNα secretion. (**A**) Immunoblot analysis of sorted follicular B (FoB) cells (CD19^+^, AA4.1^-^, CD23^+^ CD21^+^), Marginal Zone B (MzB) cells (CD19^+^, AA4.1^-^, CD23^-^, CD21^+^) and plasmacytoid dendritic cells (pDCs) (B220^+^, Siglec H^+^) to examine levels of MZB1 protein expression in each cell type. GAPDH is used as a loading control. (**B**) Immunoblot analysis of MZB1 protein level in splenic pDCs at various time points during a 24 h stimulation (image is representative of two experiments) with GAPDH used as a loading control. Uncropped blots are shown in Supplementary Fig. S3A, B. (**C**) ELISA-based quantification of secreted IFNα and IFNβ levels in the supernatants of 10^5^ pDCs from *Mzb1*^+/+^ and *Mzb1*^*−/−*^ mice at 24 h post stimulation with CpG A. Each dot represents an individual culture of sorted pDCs derived from the bone marrow of an individual mouse. (**D**) ELISA-based quantification of secreted TNFα and IL6 levels in the supernatant of 10^5^ sorted pDCs at 24 h post stimulation with CpG B. (**E**) Secreted IFNα levels, quantified by ELISA, in the supernatant of 10^5^ sorted *Mzb1*^+/+^ and *Mzb1*^*−/−*^ pDCs at 24 h post stimulation with the TLR7 agonist Imiquimod. ns, non-significant. (**F**) Flow cytometry analysis of the surface expression of the activation marker CD86 on unstimulated and CpG A-stimulated pDCs. (**G**) Box plot of quantitative RT-PCR analysis to determine the relative mRNA levels of *Ifna* in *Mzb1*^+/+^ and *Mzb1*^*−/−*^ pDCs at 6 and 24 h post stimulation with CpG A, normalized to 18S rRNA levels. The box borders represents the interquartile range and the horizontal line in the box is the median. Numbers of independent samples (n) are indicated below the graph. (**H**) qRT-PCR analysis of *Tnfa* (TNFα) and *Il6* (IL6) transcript levels in *Mzb1*^+/+^ and *Mzb1*^*−/−*^ pDCs stimulated with CpG A for 24 h. Transcript levels were normalized to those of 18S rRNA. Error bars show SD. Statistical difference between the mean was analysed by an unpaired two-tailed Student’s t-test. (***) P ≤ 0.0005. ns, non-significant.

By immunoblot analysis of sorted splenic pDCs, we found that unstimulated pDCs express MZB1 (Fig. 1A). The level of MZB1 protein expression in pDCs was similar to that found in marginal zone B cells (MzB) and higher than that detected in follicular B cells (FoB). However, we could not determine whether MZB1 is uniformly expressed in pDCs because the anti-MZB1 antibody could not be used for intracellular flow cytometry. Treatment of wild type pDCs with a TLR9 agonist, CpG A oligonucleotide (ODN) that induces high IFNα production and only weakly stimulates NF-κB signaling^32,33^, did not change the level of MZB1 protein (Fig. 1B). To assess the function of MZB1 in pDCs, we first determined the numbers and frequencies of pDCs in *Mzb1*^*−/−*^ mice. Flow cytometric analysis of Siglec H- and B220-double positive splenic pDCs indicated that their numbers and frequencies are similar in wild type and *Mzb1* knockout mice (Supplementary Fig. S1A). Because the numbers of splenic pDCs are quite low, we expaned bone marrow-derived pDCs in culture with Flt3 ligand and sorted them by using the pDC markers Siglec H and B220 (Supplementary Fig. S1B). Stimulation of MZB1-deficient pDCs with CpG A resulted in an impaired IFNα secretion relative to the wild type pDCs, as determined by an Enzyme-Linked ImmunoSorbent Assay (ELISA) (Fig. 1C). In contrast, the secretion of IFNβ, which is expressed at a much lower level than that of IFNα, was unaffected by the deletion of *Mzb1*. To examine whether MZB1 is also required for the secretion of the pro-inflammatory cytokines IL6 and TNFα, we stimulated wild type and *Mzb1*^*−/−*^ pDCs with CpG B, which activates TLR9 dependent NF-κB signaling^32^. The secretion of these cytokines, which were detected at a much lower level than that of IFNα, were not affected by the deletion of *Mzb1* (Fig. 1D). Interestingly, stimulation *Mzb1*^+*/*+^ and *Mzb1*^*−/−*^ pDCs with the TLR7 agonist Imiquimod, which is a weaker stimulator of IFNα secretion, resulted in a similar low level of IFNα secretion in wt and mutant cells (Fig. 1E). Taken together, these results suggest that MZB1 is required in conditions of very abundant secretion of IFNα.

### MZB1-deficient pDCs have a normal TLR9 signaling response

MZB1 is a co-chaperone of GRP94, which is required for the proper expression and function of various TLRs^28,34^. Therefore, we examined whether the reduced IFNα secretion reflects a defect in TLR9 signaling. Stimulation of *Mzb1*^+*/*+^ and *Mzb1*^*−/−*^ pDCs with CpG A resulted in similarly enhanced cell surface expression of the activation marker CD86, as determined by the flow cytometry analysis (Fig. 1F). Moreover, proper TLR9 signaling in *Mzb1*^*−/−*^ pDCs was confirmed by the activation of integrin β1 (CD29), which was detected in flow cytometric analysis with an antibody that recognizes the extended conformation of CD29^29^ (Supplementary Fig. S1B). Finally, the stimulation of *Mzb1*^+*/*+^ and *Mzb1*^*−/−*^ pDCs with CpG A resulted in similar increase in the levels of *Ifna* transcripts between 6hrs and 24 h after stimulation (Fig. 1G). As many different subtypes of IFNα exist, we used in this experiment degenerate primers that detect transcripts of multiple IFNα subtypes (Fig. 1G). However, we also confirmed the normal increase of *Ifna* transcripts in stimulated *Mzb1*^*−/−*^ pDCs by using specific primers for transcripts of the IFNα1–2 subtypes (Supplementary Fig. S1C).

The marked up-regulation of IFNα expression involves a feed-forward loop in which IFNα augments its own expression by binding the IFN cell surface receptor (IFNAR) and activating the JAK-STAT pathway and the expression of IFN-stimulated genes^35^. Therefore, we also examined whether the observed IFNα secretion defect of stimulated *Mzb1*^*−/−*^ pDCs is due to an impaired IFNα receptor signaling. A blockade of IFNAR signaling by an anti-IFNAR antibody has previously been shown to reduce IFNα secretion^36^. However, flow cytometric analysis to detect the surface expression of the IFNAR1 subunit did not reveal any significant differences between *Mzb1*^+*/*+^ and *Mzb1*^*−/−*^ pDCs (Supplementary Fig. S1D). Moreover, the internalization of the IFNAR1, triggered by the binding of IFNα, was not affected by the deletion of *Mzb1* (Supplementary Fig. S1E). The transcript levels of the IFNα-stimulated *Tnfa* and *Il6* genes also increased to similar levels in wild type and MZB1-deficient pDCs upon stimulation with the CpG A (Fig. 1H). Taken together, these results show that the reduced secretion of IFNα in stimulated *Mzb1*^*−/−*^ pDCs is neither due to a defective transcriptional response to TLR9 signaling nor to a defect in the IFNα feed-forward loop.

### *Mzb1*^*−/−*^ pDCs show a reduced ER expansion and an intracellular retention of IFNα

**Figure 2:**
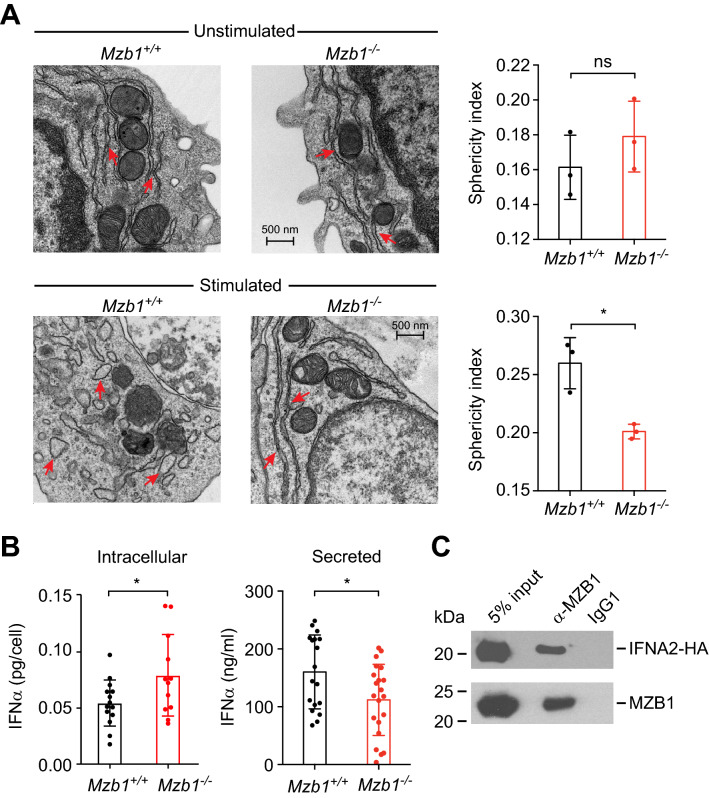
*Mzb1*^*−/−*^ pDCs show a reduced expansion of the ER and intracellular retention of IFNα. (**A**) Electron microscopy (EM) analysis of *Mzb1*^+*/*+^ and *Mzb1*^*−/−*^ pDCs that were unstimulated or stimulated with CpG A for 24 h (left-hand panels). Red arrows indicate the ER. The quantification of the sphericity index of the ER, as the ratio of the width to the length of the ER, is shown in the right-hand panels. Each dot is a biologically separate sample with approximately 50 different cells imaged for each sample. Statistical difference between the mean was analysed by an unpaired two-tailed Student’s t-test. *P ≤ 0.05. Error bars show SD. ns, non-significant. (**B**) Intracellular and secreted IFNα levels of CpG A-stimulated (24hrs) *Mzb1*^+/+^ and *Mzb1*^*−/−*^ pDCs, as determined by ELISA of lysates and supernatants of 10^5^ cells, respectively. Each dot represents pDCs derived from the bone marrow of individual mice. Data from two separate experiments were included in the analysis. Statistical difference between the mean was analysed by an unpaired two-tailed Student’s t-test. *P ≤ 0.05, **P ≤ 0.005. Error bars show SD. (**C**) Co-immunoprecipitation assay to detect the interaction of MZB1 with IFNA2. Lysates of K46 B cells expressing HA-tagged IFNA2 were incubated with an anti-Mzb1 monoclonal antibody or an IgG control. MZB1 and IFNA2 were detected by immunoblot analysis with MZB1- and HA-specific antibodies, respectively. Uncropped blots are shown in Supplementary Fig. S3C.

During an unfolded protein response (UPR), the morphology of the ER undergoes an ATF6/XBP1-driven expansion of ER morphology^20,21^. ER dilation and changes in ER morphology have also been used to assess the ER stress status of cells^37–39^. To examine whether the impaired IFNα secretion in stimulated *Mzb1*^*−/−*^ pDCs could reflect a reduced ER dilation, we evaluated the status of the ER by electron microscopy (EM) in control and stimulated conditions. The shape and morphology of the peripheral ER showed a similar structure of sheets in unstimulated *Mzb1*^+*/*+^ and *Mzb1*^*−/−*^ pDCs (Fig. 2A). After 24 h of stimulation, the ER of *Mzb1*^+*/*+^ pDCs showed marked changes in morphology, whereas the *Mzb1*^*−/−*^ pDCs maintained the elongated sheet structure (Fig. 2A). To quantify the changes in ER morphology, we calculated the sphericity indexes from the data of three independent experiments and determined that the sphericity of the ER in stimulated *Mzb1*^*−/−*^ pDCs is approximately 30% lower than that of stimulated *Mzb1*^+*/*+^ pDCs (Fig. 2A).

The reduced sphericity index of stimulated *Mzb1*^*−/−*^ suggests an impaired ER dilation and protein folding capacity. To examine whether the reduced IFNα secretion in *Mzb1*^*−/−*^ pDCs reflects an accumulation of intracellular IFNα, we quantified the intracellular levels of IFNα by performing an ELISA with lysates from wild type and *Mzb1*^*−/−*^ pDCs. A significantly higher level of intracellular IFNα was detected in *Mzb1*^*−/−*^ pDC lysates as compared to *Mzb1*^+*/*+^ pDC lysates, suggesting that the *Mzb1* deletion leads to a partial retention of IFNα in the ER (Fig. 2B). Conversely, the ELISA of the supernatants collected prior to cell lysis showed a reduced IFNα secretion by *Mzb1*^*−/−*^ pDCs relative to that by *Mzb1*^+*/*+^ pDCs.

In antibody-secreting cells, MZB1 interacts with the immunoglobulin (Ig) μ heavy chain and enhances the secretion of IgM by acting as a co-chaperone of the substrate specific GRP94 chaperone^26–29^. Therefore, we examined whether MZB1 interacts with IFNα. As the anti-IFNα antibodies did not work in the immunoblot analysis, we examined an interaction in K46 B cells that had been transfected with an HA-tagged IFNA2 expression plasmid. We found that the HA-tagged IFNA2 is co-immunoprecipitated with an anti-MZB1 antibody (Fig. 2C). These results indicate that the ER-localized co-chaperone MZB1 interacts with IFNα and may enhance its proper folding in conditions of abundant secretion of IFNα.

### *Mzb1*^*−/−*^ pDCs have a reduced ER stress response and impaired ATF6 activation

**Figure 3:**
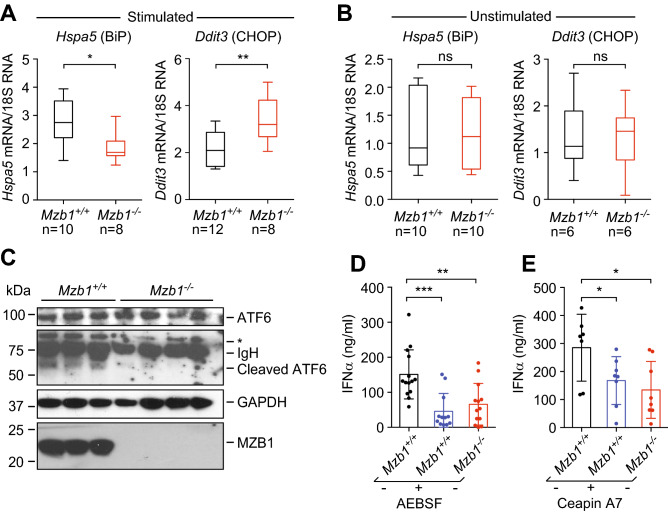
*Mzb1*^*−/−*^ pDCs exhibit an improper ER stress response due to the impaired activation of the ATF6 pathway. (**A**) Box plot of qRT-PCR analysis to determine the relative *Hspa5* (BiP) and *Ddit3* (CHOP) mRNA levels after stimulation of *Mzb1*^+*/*+^ and *Mzb1*^*−/−*^ pDCs with CpG A for 24 h. mRNA levels were normalized to those of 18S rRNA. Numbers of independent samples (n) are indicated below the graph. (**B**) qRT-PCR analysis of *Hspa5* (BiP) and *Ddit3* (CHOP) mRNA at basal levels in unstimulated pDCs. (**C**) Immunoblot analysis to detect uncleaved and cleaved ATF6 (approx. 55 kDa) in CpG A-stimulated pDCs. GAPDH serves as a loading control. IgH indicates residual IgH from the serum and * indicates an unknown cross-reacting protein. Uncropped blots are shown in Supplementary Fig. S3D. (**D**) ELISA-based quantification of the secreted IFNα in the supernatant of 10^5^ *Mzb1*^+*/*+^ and *Mzb1*^*−/−*^ pDCs that were untreated or treated with the ATF6 inhibitor AEBSF at 100 µM. (**E**) ELISA-based quantification of secreted IFNα in the supernatant of *Mzb1*^+*/*+^ and *Mzb1*^*−/−*^ pDCs, untreated or treated with the ATF6 inhibitor Ceapin A7 at 3 µM. Each dot represents an individual pDC culture derived from an individual mouse. Statistical difference between the mean was analyzed by an unpaired two-tailed Student’s t-test. *P ≤ 0.05; **P ≤ 0.005. Error bars show SD. ns, non-significant.

To further examine the molecular basis for the expansion of the ER and the impaired secretion of IFNα, we examined the UPR in wild type and *Mzb1*^*−/−*^ pDCs. In response to ER stress, the ATF6 pathway has been implicated in the up-regulation of the *Hspa5* (encoding BiP) during the early phase of the UPR^12^. Analysis of the RNA expression of *Hspa5* by quantitative RT-PCR revealed a weak up-regulation in stimulated *Mzb1*^*−/−*^ pDCs, relative to stimulated *Mzb1*^+*/*+^ pDCs (Fig. 3A). No difference in *Hspa5* expression was observed in unstimulated *Mzb1*^+*/*+^ and *Mzb1*^*−/−*^ pDCs (Fig. 3B). Thus, the diminished upregulation of BiP may lead to continued ER stress, activation of PERK pathway and increased expression of CHOP. Indeed, the level of *Ddit3* (encoding CHOP) mRNA expression in *Mzb1*^*−/−*^ pDCs was found to be higher than that in wild type pDCs, suggesting a problem in alleviating the ER stress by a proper UPR in the MZB1-deficient cells (Fig. 3A,B).

Interestingly, the expanded ER morphology observed in stimulated *Mzb1*^+*/*+^ pDCs is reminiscent of the ER morphology seen in CHO cells that overexpress the cleaved and activated transcription factor ATF6^21^. Therefore, we examined the possibility that MZB1 is involved in the activation of ATF6 under ER stress conditions. Immunoblot analysis indicated that stimulated *Mzb1*^*−/−*^ pDCs have modestly reduced levels of full-length ATF6 but significantly lower levels of the ~ 55kD cleaved form of ATF6 relative to stimulated wild type pDCs (Fig. 3C). Moreover, we examined whether or not the pharmacological inhibition of ATF6 cleavage in stimulated wild type pDCs would result in an impaired IFNα secretion. To this end, we used the serine protease inhibitor AEBSF, which inhibits the first step of ATF6 cleavage in the Golgi^40^, and Ceapin A7, which blocks the transport of ATF6 to the Golgi^41,42^. Notably, the treatment of wild type pDCs with either inhibitor showed a similar reduction in IFNα secretion as observed in *Mzb1*^*−/−*^ pDCs (Fig. 3D,E). Finally, we examined whether the MZB1 deficiency affects the IRE1 pathway and detected no obvious differences in the spliced *Xbp1* levels between stimulated wild type and *Mzb1*^*−/−*^ pDCs (Supplementary Fig. S1F). Taken together, our data suggest that *Mzb1*^*−/−*^ pDCs have a reduced ability of activating the ATF6 pathway which may account for the reduced *Hspa5* (BiP) expression and the impaired expansion of the ER.

### *Mzb1*^*−/−*^ pDCs have an impaired ability to stimulate plasma cell differentiation

**Figure 4:**
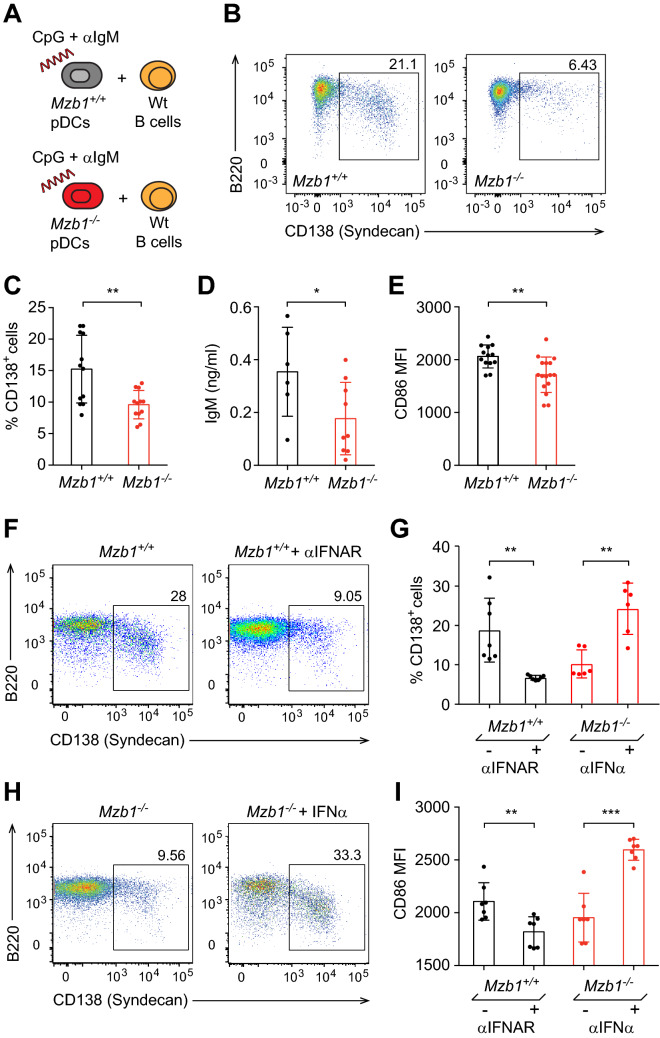
Impaired B cell differentiation towards plasma cells upon co-culture with *Mzb1*^*−/−*^ pDCs. (**A**) Schematic representation of co-cultures of MACS-sorted wild type CD19 B cells together with *Mzb1*^+*/*+^ or *Mzb1*^*−/−*^ pDCs that were stimulated with both CpG C and anti-IgM for 72 h. (**B**) Flow cytometric analysis of the co-cultures 72 h post stimulation for plasmablast markers B220 and CD138 (Syndecan). (**C**) Quantification of percentage of CD138^+^ cells. (**D**) ELISA-based quantification of secreted IgM after co-culturing CD19^+^ B cells with pDCs for 72 h. (**E**) Quantification of the mean fluorescence intensity (MFI) of the B cell activation marker CD86 by flow cytometric analysis. (**F**) Flow cytometric analysis of plasma cell differentiation in co-cultures of CD19^+^ B cells with *Mzb1*^+*/*+^ pDCs in the absence or presence of IFNAR blocking antibody. (**G**) Quantification of the frequencies of plasmablasts in co-cultures of CD19^+^ B cells with *Mzb1*^+*/*+^ pDCs with or without the IFNAR blocking antibody (black) and in co-cultures of B cells and *Mzb1*^*−/−*^ pDCs with or without exogenous IFN⍺ (red). (**H**) Flow cytometric analysis of plasma cell differentiation in co-cultures of B cells with *Mzb1*^*−/−*^ pDCs in the presence or absence of exogenous IFN⍺. (**I**) Quantification of the MFI of the activation marker CD86 in B cells, co-cultured with *Mzb1*^+*/*+^ pDCs in the presence or absence of IFNAR blocking antibody (black) and co-cultured with *Mzb1*^*−/−*^ pDCs with or without exogenous IFN⍺ (red). Each dot represents co-cultures with pDCs derived from an individual mouse. Statistical difference between the mean was analyzed by an unpaired two-tailed Student’s t-test. *P ≤ 0.05 **P ≤ 0.005. Error bars show SD.

IFNα and pDCs play a role in enhancing humoral immunity by activating B and T cells^43,44^. To explore the functional consequences of the reduced IFNα secretion by *Mzb1*^*−/−*^ pDCs on B cell activation, we performed co-culture experiments. MACS-sorted wild type CD19-positive B cells were co-cultured with either *Mzb1*^+*/*+^ or *Mzb1*^*−/−*^ pDCs. The cultures were stimulated with CpG C and anti-IgM for 72 h (Fig. 4A). Flow cytometric analysis indicated that the percentage of CD138-positive plasmablasts is lower in the co-culture with *Mzb1*^*−/−*^ pDCs as compared to the co-cultures with *Mzb1*^+*/*+^ pDCs (Fig. 4B,C). By performing ELISA with cell culture supernatants, we also detected lower levels of IgM in the co-cultures with the *Mzb1*^*−/−*^ pDCs relative to the co-cultures with the wild type pDCs (Fig. 4D). Moreover, we observed a reduced surface expression of the CD86 activation marker on B cells that were co-cultured with *Mzb1*^*−/−*^ pDCs (Fig. 4E).

To determine whether the impaired stimulation of plasma cell differentiation by *Mzb1*^*−/−*^ pDCs is a result of the reduced IFNα secretion, we included an IFNAR blocking antibody in co-cultures of *Mzb1*^+*/*+^ pDCs to reduce the secreted IFNα levels. In this experiment, we also observed an impaired plasmablast differentiation (Fig. 4F,G). Conversely, the reduced differentiation of plasmablasts in co-cultures with *Mzb1*^*−/−*^ pDCs could be rescued by the addition of exogenous IFNα (Fig. 4G,H). The effects of altered IFNα levels on plasmablast differentiation were also reflected by equivalent changes in the surface expression of CD86 (Fig. 4I). Thus, the reduced ability of IFNα secretion in *Mzb1*^*−/−*^ pDCs results in an impaired stimulation of B cell differentiation towards antibody-secreting plasmablasts.

## Discussion

pDCs are well known for their capacity to secrete high levels of IFNα after TLR9 stimulation during viral infections. High levels of protein secretion lead to increased ER stress in these cells and result in the activation of the unfolded protein response (UPR). We and others have previously described the role of MZB1 as an ER resident co-chaperone of GRP94 that enhances the secretion of IgM and IgA and promotes plasma cell differentiation^25,26,28,45^. Here we show that MZB1 is expressed in pDCs and is required for abundant IFNα secretion, which is a major hallmark of pDCs. MZB1 is a member of a small family of CNPY co-chaperones that interact with GRP94^34,46^. In particular, CNPY3, which is ubiquitously expressed and localized in the ER, acts a GRP94 co-chaperone that enhances the folding of TLR4^34^. Therefore, we considered the possibility that MZB1 affects the function of TLR7 and/or TLR9. In MZB1-deficient pDCs, however, we observed a normal TLR9 signaling response, as evidenced by the similar transcriptional activation of the *Ifna* genes in mutant and wild type pDCs. Moreover, in *Mzb1*^*−/−*^ pDCs we detected higher amounts of intracellular IFNα as compared to *Mzb1*^+*/*+^ pDCs, suggesting that IFNα is produced but not properly secreted, possibly due to an impaired protein folding in the ERIn antibody-secreting B cells, MZB1 has been shown to augment the interaction of the Ig μ heavy chain with the chaperone GRP94 in conditions of abundant Ig expression^28^, and therefore, the observed interaction of MZB1 with IFNα suggests a similar mechanism of MZB1 function in pDCs. According to this view, the interaction of MZB1 with IFNα occurs in the ER and enhances the folding of IFNα in conditions of abundant protein expression and ER stress. In contrast, the function of MZB1 is not required for the weak IFNα secretion in Imiquimod-stimulated pDCs, in which the expression of IFNα is only modestly enhanced relative to unstimulated pDCs.

Inefficient protein folding can be the result of an impaired ER stress response. Indeed, we observed two hallmarks of unresolved ER stress. First, we found that *Mzb1*^*−/−*^ pDCs do not upregulate the major protein folding chaperone BiP. In addition, the EM analysis of stimulated pDCs indicated that *Mzb1*^*−/−*^ pDCs do not acquire the dilated ER morphology detected in *Mzb1*^+*/*+^ pDCs. Both features of ER stress have been linked to the ATF6 pathway of the UPR^12,21,47^. In particular, the activation of ATF6 involves the dissociation of BiP, translocation to the Golgi, proteolytic cleavage and subsequent nuclear translocation is responsible for the transcriptional upregulation of the chaperone genes^16,18,19^. Moreover, changes in the abundance of activated ATF6 by overexpression have been linked to changes in ER morphology^21^. Therefore, we propose a model in which MZB1 enhances the interaction of BiP with the client IFNα, resulting in a default liberation of ATF6 from BiP as an initial step in its activation (Supplementary Fig. S2A,B). CNPY2, a ubiquitously expressed member of the CNPY family of co-chaperones, has been shown to initiate the PERK pathway of the UPR^48^. In contrast, MZB1 is selectively expressed in cells with a high secretory demand, which may enable these cells to reach their full secretory capacity. Indeed, all known roles for MZB1 have been linked to the efficient secretion and protein folding in conditions of ER stress.

pDCs have been known to play a role in linking innate and adaptive immunity. Type I interferons have been shown to enhance humoral immunity and promote immunoglobulin isotype switching^49^. Moreover, CpG-stimulated pDCs induce plasma cell differentiation in naïve B cells^50^. Indeed, we find that the differentiation of B cells towards plasma cells is augmented by the co-culturing of B cells with stimulated wild type pDCs. In *Mzb1*^*−/−*^ pDCs, however, the reduced IFNα secretion resulted in diminished differentiation towards plasma cells and in lower levels of IgM secretion. The function of MZB1 in pDCs could be relevant during chronic viral and microbial infections in which the IFNα secretion enables an innate immune response and stimulates B cells to differentiate into antibody-secreting plasma cells. Furthermore, IFNα plays a crucial role in autoimmune diseases like psoriasis and SLE^3,8^. In murine psoriasis xenograft models in which pDCs infiltrate the skin, the inhibition of IFNα secretion has been found to ameliorate the disease symptoms. In SLE, MZB1 expression is frequently upregulated^51^, which could account for the enhanced secretion of IFNα and the augmented secretion of auto-antibodies by B cells. The increased IFNα levels in SLE patients as a driver of disease progression also correlate with the reduced frequency of immunosuppressive regulatory B cells (Breg)^52^. Clinical studies have shown that blocking type I interferons helps to alleviate SLE symptoms and disease progression. MZB1 has a more restricted expression pattern than IFNα, which makes it an attractive potential therapeutic target in autoimmune diseases.

## Materials and methods

### Cell culture and cell isolation

Bone marrow cells from mice were prepared following the standard protocol. Cells were counted using the Casy Cell Counter and suspended at 2 × 10^6^ cells/ml in RPMI with 10% FCS, Penicillin, Streptomycin, 2 mM L-Glutamine and beta-mercaptoethanol, supplemented with 200 ng/ml Flt3 ligand. On day 8, fully developed pDCs were sorted by flow cytometry for the surface expression of both B220 and Siglec H. Cells of the K46 B cell line were transfected with an Ifna2-pMys-expression plasmid in which an HA-tag is attached to the C-terminus of IFNa2. Splenic pDCs were MACS-sorted with a mouse Plasmacytoid dendritic cell isolation kit (Miltenyi Biotech) and subsequently sorted by flow cytometry FACS.

### Cloning and retroviral transduction

For the expression of HA-tagged IFNA2 protein, *Ifna2* cDNA was cloned with an optimized Kozak sequence at the 5′ end and HA-tag sequence at the 3′end into a pMYs-IRES-GFP bicistronic vector to allow for the expression of IFNA2 along with GFP. For the production of HA-IFNA2-expressing retroviruses, platE cells were transfected with the expression plasmid. Supernatant of transfected Plat E cell was used for transduction of K46 B cells and GFP-expressing cells were sorted prior to the preparation of cell lysates for co-immunoprecipitation.

### CpG oligonucleotide-mediated stimulation, inhibition of ATF6 cleavage and B cell co-cultures

Sorted bone marrow-pDCs were aliquoted into 96 well plates at 100,000 cells per well. Cells were stimulated with 1 mM of CpG A (ODN1585) or B (ODN1886) for 24 h, unless otherwise stated. For the pharmacological inhibition of the ATF6 pathway, cells were treated with AEBSF at a final concentration of 100 µM or with Ceapin A7 (Sigma) at a concentration of 3 µM, four hours post stimulation with CpG ODN. For co-culture experiments 100,000 CD19-positive wild type B cells, purified by a positive MACs selection, were co-cultured with 30,000 FACS-sorted bone marrow-derived pDCs and stimulated with 1 mM CpG C (ODN M362) and anti-IgM for 72 h as previously described in^50^. All ODNs used were obtained from Invivogen.

### RNA isolation and RT-PCR

RNA was isolated following homogenisation in Trizol according to the manufacturer’s instructions. cDNA was reverse transcribed using Thermo Fischer cDNA kit. RT-PCR analysis was carried out using Sybr green reagents. Primers used are listed in the Supplemental Information.

### Cytokine quantification by enzyme-linked immunosorbent assay (ELISA)

Measurements of the cytokines IFNα, IFNβ, IL6 and TNFα were performed by ELISA. The following kits were used according to the manufacturer’s protocol. Mouse IFN-alpha Platinum ELISA-4, Mouse IL-6 Ready-SET-go, Mouse TNF-alpha Ready-Set-go (eBiosciences) and LegendMax Mouse IFNβ ELISA kit (BioLegend).

### Immunoprecipitations and immunoblot analysis

Cells were resuspended in 20 mM HEPES pH7.6, 1 × Protease inhibitor mix, PMSF, 0.1% NP40, 10 mM Sodium orthovanadate, 2 mM magnesium chloride, 150 mM Sodium chloride and 10% glycerol, followed by three sonications for 30 secs. The protein concentrations were measured by Bradford assays, and the samples were diluted with Laemmli buffer and run on 4–12% SDS PAGE gels. The proteins were transferred onto a membrane and probed with the appropriate antibodies.

Immunoprecipitations of MZB1 were carried out by using Protein-G Dyna-beads which were blocked overnight with 1% BSA. Following a 4-h immunoprecipitation at 4 °C, the beads were washed with the lysis buffer five times. After gel electrophoresis, the samples were visualized by immunoblot analysis with antibodies listed in the Supplemental Information. Co-immunoprecipitation experiments to examine the interaction between MZB1 and HA-tagged INFA2 in transfected K46 B cells were performed by treating cells with a BD Golgi stop (4 µl/6 ml culture medium) for 5 h and then lysed in the buffer described above.

### FACS analysis

Single-cell suspensions were resuspended in PBS with 2% FCS and stained for flow cytometric analysis. Antibodies used are listed in the Supplemental Information. Data were acquired with an LSR Fortessa (BD Biosciences) and analyzed using the Flow jo software (version 10.1r5; https://www.flowjo.com/solutions/flowjo/downloads/previous-versions).

### Electron microscopy

Cells were pelleted and resuspended in 2.5% glutaraldehyde in 0.1 M sodium cacodylate buffer pH 7.4 and incubated for 20 min at RT. Subsequently, reduced IFNα secretion capacity he cells were pelleted and resuspended in 0.1 M sodium cacodylate pH7.4 and stored at 4 °C for further processing. After dehydration, the samples were embedded in Eponate 12 resin (Ted Pella) and sections were cut. Images were acquired using a FEI Tecnai12 Transmission electron microscope equipped with a TIETZ digital camera. ER enlargement was analyzed by sphericity index. Briefly, major and minor axis were measured for each ER compartment identified in 50 randomly selected images per condition. Sphericity index was calculated according to the formula major/minor axis.

### Mouse lines and in vivo experiments

All mouse experiments were carried out in accordance to the guidelines of the Federation of European Laboratory Animal Science Association and following legal approval of the animal committee of the government bureau (Regierungspräsidium) Freiburg, Germany (license Gr-iTO-1). *Mzb1*^+*/*+^ and *Mzb1*^*−/−*^ mice were generated as previously described^28^. Mouse strains were bred and maintained in the animal care facility of the Max Planck Institute of Immunobiology and Epigenetics. Experiments were performed with 7 to 14-week-old mice in backcrossed SV129 background.

## Supplementary Information

Supplementary Information
