# Supplementary material for: MZB1 enables efficient interferon α secretion in stimulated plasmacytoid dendritic cells
Source: Sci Rep. 2020 Dec 14;10:21626. doi: 10.1038/s41598-020-78293-3 (PMC7736851; doi:10.1038/s41598-020-78293-3)
Supplement: Supplementary file 1 — Supplementary Information [file 41598_2020_78293_MOESM1_ESM.pdf]

Supplementary Information for

MZB1 enables efficient interferon α secretion in stimulated plasmacytoid dendritic cells

Tanya Kapoor, Mauro Corrado, Erika L. Pearce, Edward J. Pearce and Rudolf Grosschedl

Corresponding author: Rudolf Grosschedl (grosschedl@ie-freiburg.mpg.de)

This PDF file includes:

Figure S1
Figure S2
Figure S3
Table S1
Table S2

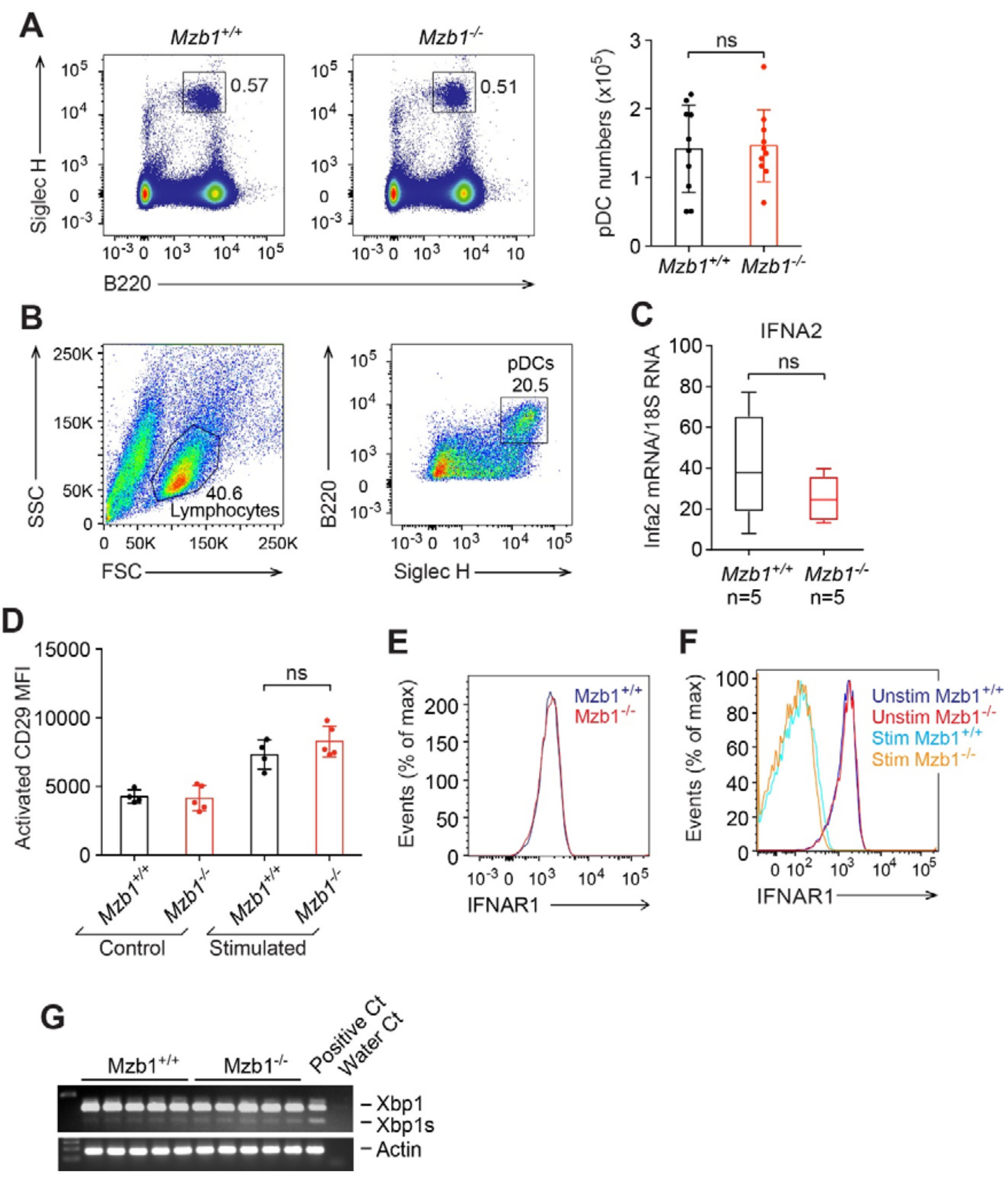

Figure S1 Related to Fig. 1 (**A**) Flow cytometric analysis of the frequency of splenic pDCs (B220⁺ Siglec H⁺) in *Mzb1*$^{+/+}$ and *Mzb1*$^{-/-}$ mice and quantification of the total numbers of splenic pDCs. (**B**) Sorting strategy for bone marrow-derived pDCs. Post 8 days of culture with Flt3 ligand, cells were sorted for the pDC markers B220 and Siglec H. (**C**) Box plot of qRT-PCR analysis to determine the relative *Ifna2* mRNA levels in *Mzb1*$^{+/+}$ and *Mzb1*$^{-/-}$

pDCs 24 hrs post stimulation with CpG A ODN. *Ifna2* RNA levels were normalized to 18S rRNA. (n=5). (**D**) MFI quantification of flow cytometric analysis of the extended form of CD29 (β1 integrin) in unstimulated and CpG A ODN-stimulated pDCs. (**E**) Flow cytometric analysis of the surface expression of the IFNAR1 subunit on unstimulated pDCs. (**F**) Flow cytometric analysis of the internalization of the IFNAR1 subunit following CpG A ODN stimulation of pDCs. (**G**) Semi-quantitative RT-PCR to analyze transcript levels of unspliced *Xbp1* and spliced *Xbp1s* in CpG A ODN-stimulated pDCs. Actin is used as a loading control. Each dot represents pDCs derived from the bone marrow of individual mice. Data from ≥2 separate experiments have been included in the analysis. Statistical difference between the mean was analyzed by an unpaired two-tailed Students t-test. Error bars show SD.

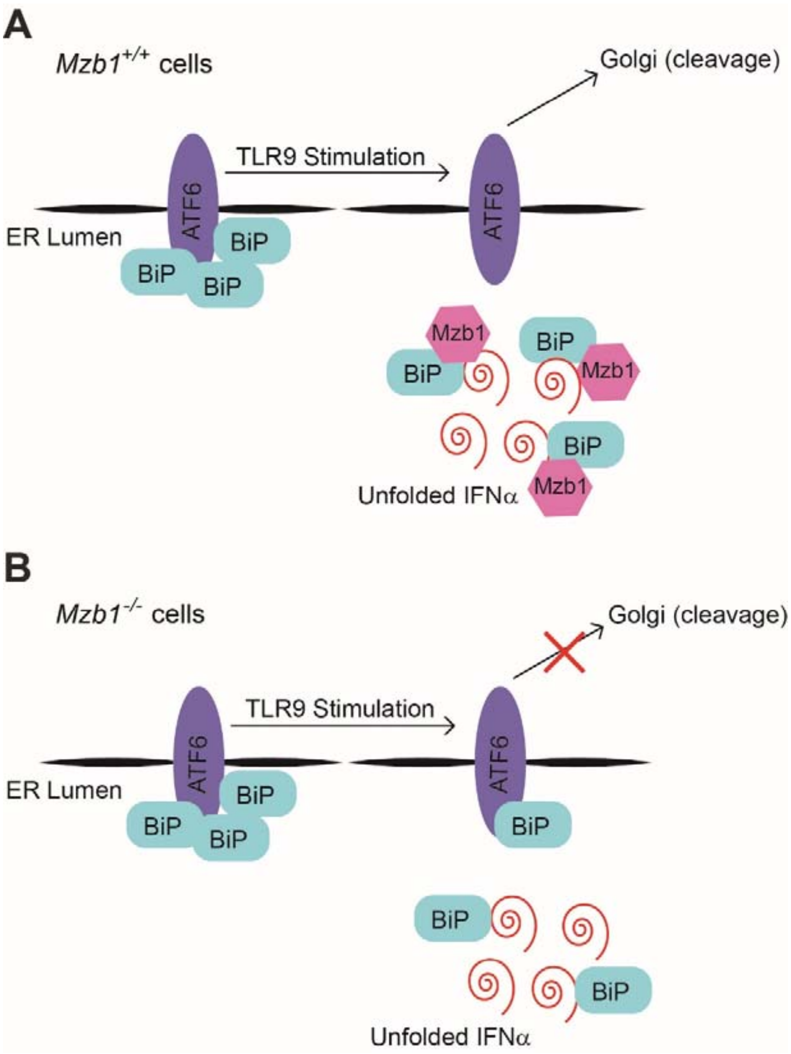

Figure S2 Proposed mechanism of MZB1 function in CpG-stimulated pDCs. (**A**) Schematic representation of the liberation of ATF6 by BiP upon TLR9 stimulation resulting in high levels of IFNα secretion and increased ER stress. In short MZB1 behaving in its capacity of a co-chaperone enhances the interaction of the chaperone BiP with its client IFNα, liberating ATF6 and allowing it to translocate to the Golgi for its subsequent cleavage and activation. (**B**) Schematic representation of the impaired activation of ATF6 upon TLR9 stimulation in *Mzb1*$^{-/-}$ cells. Reduced association of BiP with the unfolded IFNα due to the lack of MZB1 results in an impaired translocation of ATF6 to the Golgi and its subsequent activation by proteolytic cleavage. Impaired ATF6 activation results in improper dilation of the ER and reduced IFNα secretion.

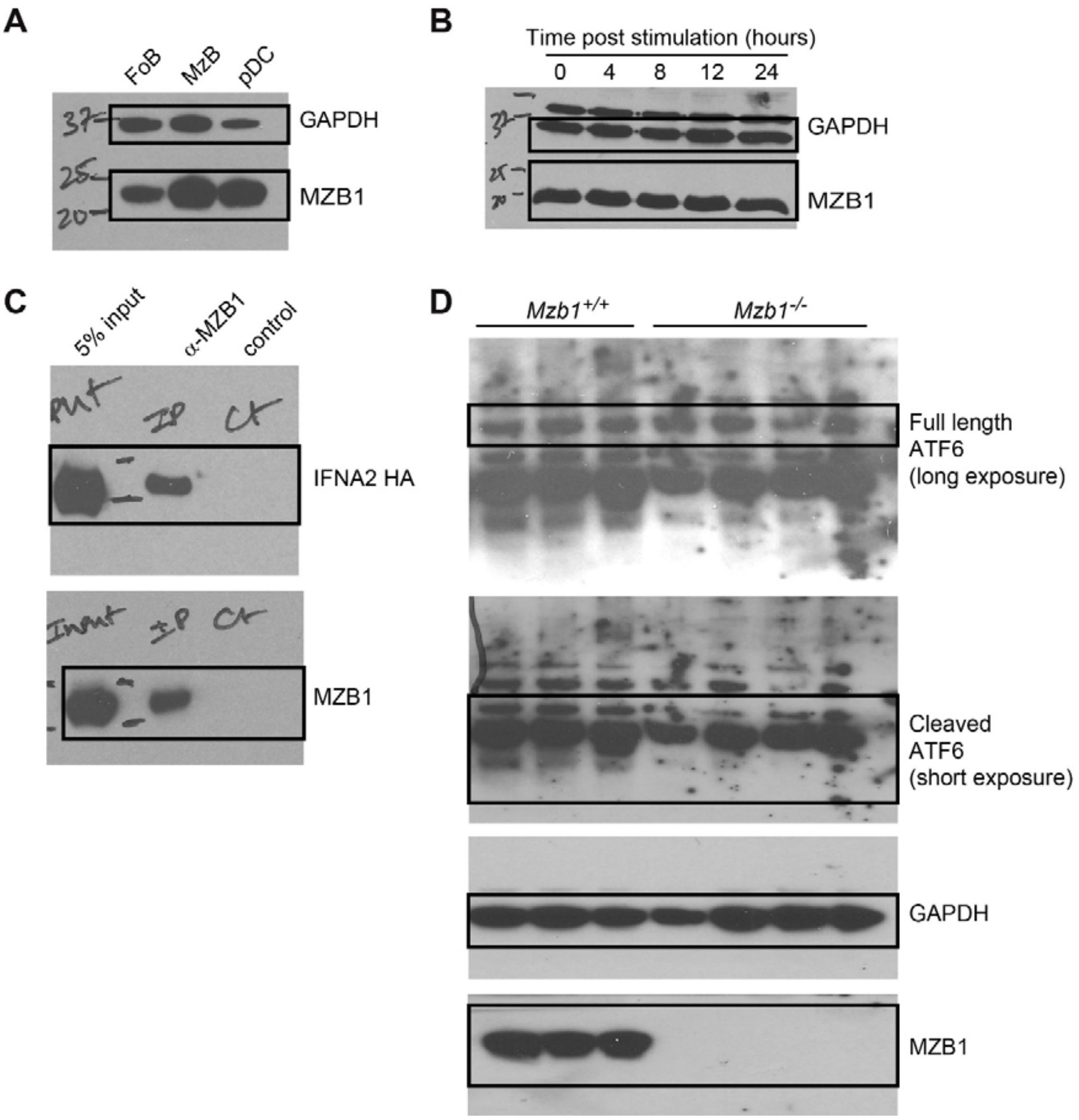

Figure S3. Uncropped Immunoblots. (A, B) Uncropped blots presented in Figures 1A and 1B. (**C**) Uncropped blots presented in Figure 2C. (**D**) Uncropped blots presented in Figure 3C. Individual panels represent different parts of a single membrane that were incubated with different antibodies for protein detection.

Table S1. qRT-PCR primer sequences.

Gene	Primer Sequence
Actin	Fwd: 5’ GTT TGA GAC CTT CAA CAC CCC 3’
	Rev: 5’ GTT TGA GAC CTT CAA CAC CCC 3’
Ddit3 (CHOP)	Fwd: 5’ GCA TGA AGG AGA AGG ACC AG 3’
	Rev: 5’ CTT CCG GAG AGA CAG ACA GG 3’
Xbp1s	Fwd: 5’ ACA CGC TTG GGA ATG GAC AC 3’
	Rev: 5’ CCA TGG GAA GAT GTT CTG GG 3’
Hspa5 (BiP)	Fwd: 5’CAT GGT TCT CAC TAA AAT GAA AGG 3’
	Rev: 5’GCT GGT ACA GTA ACA ACT 3’
IL6	Fwd: 5’CTC TGG GAA ATC GTG GAA AT 3’
	Rev: 5’CCA GTT TGG TAG CAT CCA TC 3’
TNFα	Fwd: 5’GCC TCT TCT CAT TCC TGC TTG 3’
	Rev: 5’CTG ATG AGA GGG AGG CCA TT 3’
18S rRNA	Fwd:5’CCATCC AAT CGG TAG TAG CG 3’
	Rev: 5’GTA ACC CGT TGA ACC CCA TT 3’
Ifna	Fwd: 5’GCC TTG ACA CTC CTG GTA CAA ATG AG 3’
	Rev: 5’ CAG CAC ATT GGC AGA GGA AGA CAG 3’
Ifna 1-2	Fwd: 5´- TGT CTG ATG CAG CAG GTG G -3´
	Rev: 5´- AAG ACA GGG CTC TCC AGA C -3´

Table S2. List of Antibodies.

Protein	Antibody Clones
ATF6	70B1413.1 Enzo
GAPDH	6C5 Millipore
Rat IgG2a Isotype	2A8F4 Southern Biotech
CD19 BV421	1D3 Biolegend 115538
CD23 PE-Cy7	B3B4 Biolegend 101614
CD21 FITC	7G6 BD 553818
CD93 APC	AA4.1 e-Bioscience 17-5892-83
B220 FITC	RA3-6B2 BD 552771
CD138 APC	281-2 Biolegend 142506
CD86 BV605	GL1 Biolegend 105037
Siglec H	551 Biolegend 129605
IFNAR1	MAR1-5A3 Thermo 16-5945-85
CD29	9EG7
